# Introduction of mandatory masking in health care and community: experience from Jena, Germany

**DOI:** 10.1007/s15010-023-02015-w

**Published:** 2023-04-03

**Authors:** Mathias W. Pletz, Andrea Steiner, Miriam Kesselmeier, Bettina Löffler, Jens Maschmann, Andreas Stallmach, Sabine Trommer

**Affiliations:** 1 Institute for Infectious Diseases and Infection Control, Jena University Hospital, Friedrich-Schiller-University, Am Klinikum 1, 07747 Jena, Germany; 2 Department for Occupational Health, Jena University Hospital, Jena, Germany; 3 Institute of Medical Statistics, Computer and Data Sciences, Jena University Hospital, Jena, Germany; 4 Center for Sepsis Control and Care (CSCC), Jena University Hospital, Jena, Germany; 5 Institute for Medical Microbiology, Jena University Hospital, Jena, Germany; 6 Public Health Department, City of Jena, Jena, Germany; 7 Medical Executive Board, Jena University Hospital, Jena, Germany; 8 Department of Internal Medicine IV (Gastroenterology, Hepatology and Infectious Diseases), Jena University Hospital, Jena, Germany

Mandatory masking the health care setting and in the community contributes to control the spread of SARS-CoV-2 and is recommended by the WHO (https://app.magicapp.org/#/guideline/6439), but supporting data are rare.

Two weeks after the Jena University Hospital (JUH) had implemented mandatory masking in March 2020 and presented the impact to the local public health authorities, the City of Jena was the first community in Germany to issue an order on mandatory public masking. Here we report the development of the number of novel infections in the Jena University Hospital and the city of Jena after implementation of mandatory masking in our hospital (i) and the city (ii) in the two months following the first introduction (between15 March to 15 May 2020).

For the JUH, numbers of novel infections were provided by the Department for Occupational Health, which has started with a thorough PCR based HCW screening (all HCW returning from travel, all HCW with symptoms and all contacts of novel infections) since 11th March. For the City of Jena, the City of Erfurt and the entire State of Thuringia, cumulative numbers of novel infections were taken from the Thuringian COVID-19 bulletins, which were released daily from 16th March 2020 by the Thuringian state authority. The cases registered in Germany can now be viewed on a daily basis in relation to rural and urban districts on a dashboard established by the Robert Koch Institute (https://experience.arcgis.com/experience/478220a4c454480e823b17327b2bf1d4).

Impact of mandatory masking in the Jena University Hospital (JUH): The JUH is a tertiary academic hospital with about 1400 beds and 5600 employees providing care for about 53,000 inpatients per year. It is the only hospital in Jena.

On 11th March 2020, the first COVID-19 cases were detected in Jena—travelers returning from skiing in Austria—among them many HCWs of the JUH. On 16th March, one of these HCWs caused the first nosocomial outbreak in JUH. Consequently, extensive PCR screening was conducted between 11th March and 12th May. Induced by further 31 positive cases until 19th March (Fig. 1), mandatory masking with surgical masks for all HCW involved in patient care was implemented on 20th March and routine staff screening continued. Implementation of masking was associated with a substantial drop in the rate of new infections among HCWs from 10.1% (31of 306 HCW screened by PCR) before to 0.4% (4 of 1005 HCW screened by PCR) after implementation of mandatory masking.

**Fig. 1:**
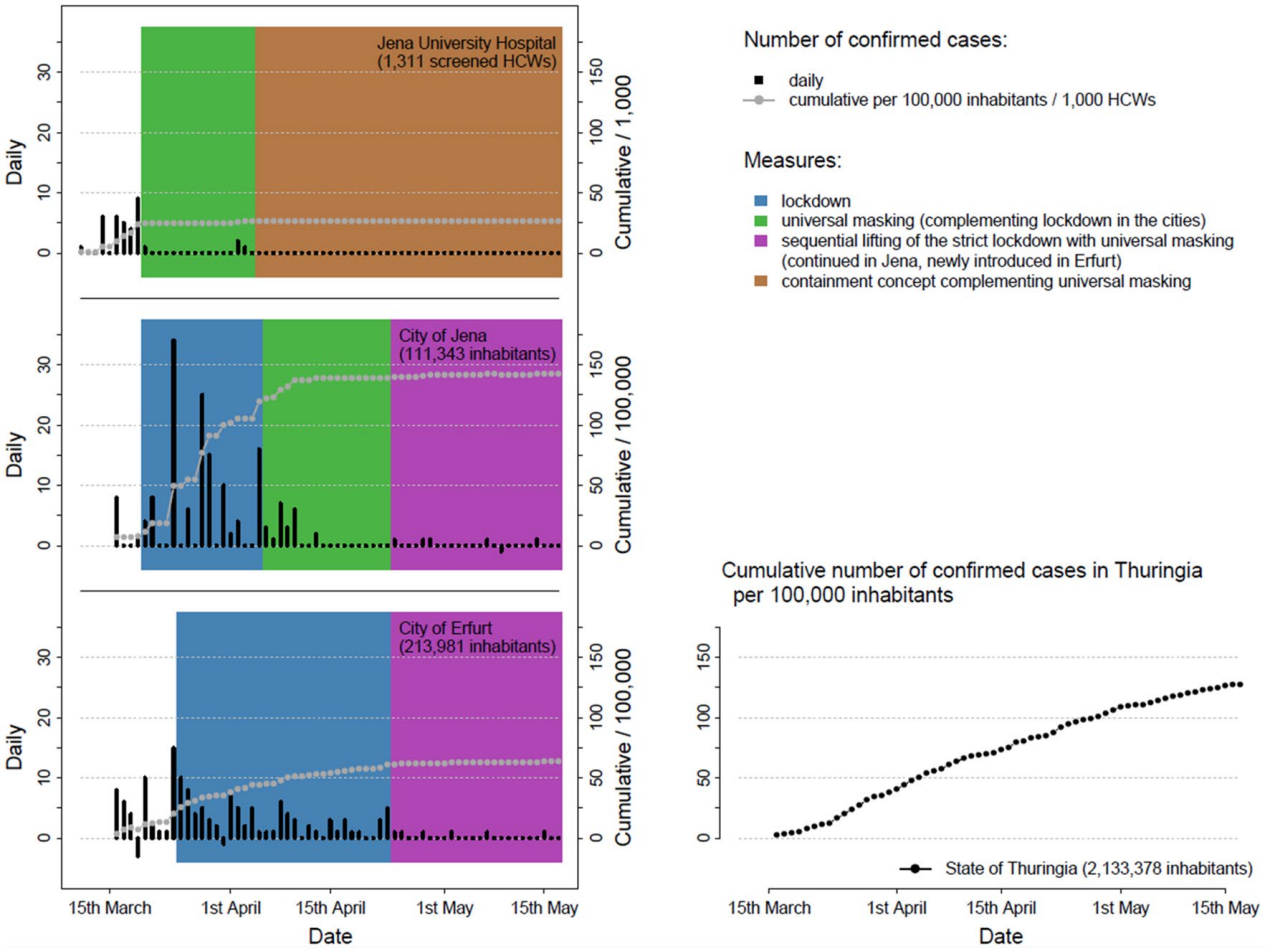
Daily reported number of novel confirmed SARS-CoV-2 infections and corresponding cumulative numbers per 1000 health care workers (HCW) of Jena University Hospital (JUH, 1500 beds) as well as per 100,000 inhabitants of the Thuringian cities Jena and Erfurt (left panel). All reported cases comprise only the first SARS-CoV-2 test results. Results of multiple testing are excluded because all infected subjects are reported by full name and address to the respective Public Health Departments. For comparison, the cumulative number per 100,000 inhabitants in the State of Thuringia is provided (right panel). The measures implemented at the JUH and in the cities are color-coded. Please note, “negative” bars for daily reported novel infections in Jena and Erfurt are explained by re-classification of individual COVID-19 cases as non-COVID-19 after quality control of the respective data and laboratory reports by the State Health Department of Thuringia. The number of inhabitants (on 31st December 2019) was taken from the Thuringian State Office of Statistics

In addition, a strict containment system was introduced on 5th April, which included placing all patients in single rooms upon admission and SARS-CoV-2 screening regardless of their admitting chief complaint. Thereafter, HCW screening did not reveal any further positive case over the here reported period.

Impact of mandatory masking in the City of Jena: Jena is the second largest city in the State of Thuringia, Germany, with approximately 111,400 inhabitants. Despite implementation of three “screening points” for patients with fever and respiratory symptoms, thorough contact tracing and isolation of confirmed COVID-19 positive cases by the authorities, a rapid increase in numbers was observed after the above mentioned first COVID-19 patient (Fig. 1). The “lockdown” (closing of stores, schools, kindergartens, churches etc.) together with social distancing measures was strictly implemented on 20th March in Jena and–with identical measures–four days later in the entire Federal State of Thuringia. As novel infections continued to occur despite the lockdown and based on the experience with mandatory masking in the JUH, Jena authorities were the first in Germany to introduce mandatory community-wide masking*, i.e.* mandatory covering of the mouth and nose in public buildings and public transport accepting also cloth masks or scarfs. The respective official order was published on 31st March to inform the population and fully applied since 6^th^ April. Since there was a shortage of surgical masks at the time, many citizens used homemade cloth masks. As shown in Fig. 1, there were no new COVID-19 cases in Jena five days after implementation, which is in line with the average incubation time of SARS-CoV-2. On 24th April, a state-wide order for community-wide masking in combination with first relaxations of the strict lockdown was issued. In Jena, four more novel cases occurred after the lifting of the lockdown within the period here reported. Three of these were health care workers that were infected at work in health care facilities outside the State of Thuringia, for the remaining patient no source of infection could be identified.

In comparison, the nearby City of Erfurt introduced mandatory masking four weeks later together with lifting of lockdown measures. Despite the lifting, the rate of novel infections was decreasing in Erfurt (from 9.4 per week and 100,000 inhabitants during the lockdown to 0.9 per week and 100,000 inhabitants after implementation of mandatory masking; Fig. 1). Erfurt was chosen as a comparison because it is the city within Thuringia that most resembles Jena. Both cities are the largest in Thuringia (Jena: 108,00 inhabitants, Erfurt: 215,500 inhabitants), both have a relatively young population compared to other regions (average age Jena 42.9 years, Erfurt 44.6 years) and both are home to a university. According to the Thuringian Social Atlas of 2020, (https://www.tmasgff.de/publikationen#38) Jena and Erfurt are considered growth centers, characterized by an increasingly academic labor market, low housing vacancy rates and a moderate population trend with a slightly growing population. The gross domestic product per person in employment in 2019 was 69,550 euros in Jena and 61,460 in Erfurt (https://www.deutschlandatlas.bund.de/). In both cities, the proportion of people without German citizenship is 7–9%.

We are aware that mere association is not causation and that our conclusions are limited by the observational nature of the data and bias factors such as additional infection control approaches (i.e. concomitant screening in the hospital and low-threshold testing services for the public), human behavior affected by masking and the public announcement of the risk for infection and the high dispersion factor associated with the spread of COVID-19.

In December 2020, a complex statistical analysis was published by economists from the University of Mainz and ETH Zurich, according to which the introduction of the mask in Jena prevented about 75% of all new infections in the following 20 days [1]. This statistic was based on the formation of synthetic controls consisting of municipalities or cities that had introduced the mask at different time points. Overall, this work showed a reduction in the daily growth rate of COVID-19 cases by approximately 47%. The authors also concluded that mandatory masking is at least as effective as pure lockdown measures and causes substantially less economic losses. Our observation, i.e. the rate of novel infections declined only moderately after the start of the lockdown but substantially after implementation of mandatory masking, is in line with this conclusion.

Surprisingly for us, the first-time introduction of the mandatory mask requirement both in our hospital and in the city of Jena always met with a high level of understanding among health care workers and citizens. In particular, the introduction in Jena was politically well prepared: the mask obligation was announced seven days in advance and in the lack of availability of paper masks, fabric masks were also acceptable at that time. Many cloth masks were produced and made available in large numbers by local businesses and citizens' initiatives. Details and background information on the implementation of the mandatory mask in the city of Jena can also be found here [2].

The value of mandatory masking in health care and the community remains an issue of debate. This is not least because individually randomized studies with a clear endpoint (e.g. detecting all transmission) and little bias to answer this question are hardly possible. Thus, mandatory masking at least partially escapes the formalized and standardized assessment of medical interventions according to evidence. It is difficult to study the effect of mandatory mask use in a randomised controlled trial for several reasons. For example, there are synergistic effects when everyone wears a mask, as it is not only about individual protection but also about inhibiting the spread of pathogens through mildly or asymptomatically infected people (source control). Therefore, most randomised trials are not randomised at the individual level but at the cluster level. In cluster randomised trials, there are again many influencing factors (e.g. infection activity in the individual clusters at different times, pre-existing immunity, compliance with the measures, risk perception, etc.).

Furthermore, there is an increasing body of evidence of side effects of mandatory masking, e.g. such as increase in contact dermatitis [3]. However, a recent meta-analysis including 31 studies with 13,329 participants showed that wearing masks was effective in preventing respiratory viral infections, i.e. SARS-CoV-1, MERS, Influenza and SARS-CoV-2. This meta-analysis also contained six RCTs, one of them on COVID-19 [4]. A 2020 meta-analysis based on 172 observational studies across 16 countries also shows that distance > 1 m (risk difference  – 10.2%, 95% CI  – 11.5 to  – 7.5), face mask (risk difference  – 14.3%, 95% CI  – 15.9 to  – 10.7) and eye protection ( risk difference  – 10.6%, 95% CI  – 12.5 to  – 7.7) significantly reduce the likelihood of SARS-CoV-2, MERS and SARS-CoV-1 infection [5].

However, the experience we present here is from the early phase of the pandemic. The Wuhan variant, which was the dominating variant at that time, was less infectious compared to later variants. Therefore, our experience from 2020 cannot be unreservedly applied to the currently circulating SARS-CoV-2 variants.

Even though the above data is from the early days of the pandemic, we believe that these are of interest. They also show that in a pandemic, the early implementation of measures can have strong productive effects. While evidence is supposed to be the basis of medical and public health decisions, there are situations in which this evidence is lacking but timely decisions still have to be made. In a pandemic in particular, speed is decisive, or as WHO executive director Mike Ryan put it: "Perfection is the enemy of the good when it comes to emergency management. Speed trumps perfection."

## Data Availability

Data on the incidence in HCW can be made available on request. Data on the incidence in Jena can be obtained by the website mentioned above and the interactive dashboard of the Robert Koch Institute (https://experience.arcgis.com/experience/478220a4c454480e823b17327b2bf1d4/page/Landkreise/).
